# Efficient non-unique probes selection algorithms for DNA microarray

**DOI:** 10.1186/1471-2164-9-S1-S22

**Published:** 2008-03-20

**Authors:** Ping Deng, My T Thai, Qingkai Ma, Weili Wu

**Affiliations:** 1 Computer Science Department, the University of Illinois at Springfield, Springfield, IL, 62703, USA; 2 Computer and Information Science and Engineering Department, University of Florida, Gainesville, FL, 32611, USA; 3 Department of Economic Crime and Justice Studies, Utica College, Utica, NY 13502, USA; 4 Computer Science Department, the University of Texas at Dallas, Richardson, TX, 75081, USA

## Abstract

**Background:**

Temperature and salt concentration are very helpful experimental conditions for a probe to hybridize uniquely to its intended target. In large families of closely related target sequences, the high degree of similarity makes it impossible to find a unique probe for every target. We studied how to select a minimum set of non-unique probes to identify the presence of at most *d* targets in a sample where each non-unique probe can hybridize to a set of targets.

**Results:**

We proposed efficient algorithms based on Integer Linear Programming to select a minimum number of non-unique probes using *d*-disjunct matrices. Our non-unique probes selection can also identify up to *d* targets in a sample with at most *k* experimental errors. The decoding complexity of our algorithms is as simple as O(*n*). The experimental results show that the decoding time is much faster than that of the methods using *d*-separable matrices while running time and solution size are comparable.

**Conclusions:**

Since finding unique probes is often not easy, we make use of non-unique probes. Minimizing the number of non-unique probes will result in a smaller DNA microarry design which leads to a smaller chip and considerable reduction of cost. While minimizing the probe set, the decoding ability should not be diminished. Our non-unique probes selection algorithms can identify up to *d* targets with error tolerance and the decoding complexity is O(*n*).

## Background

One of recent important developments in biology is the success of Human Genome Project. This project was done with a great deal of help from computer technology. The technique for obtaining sequenced genome data is getting mature. More and more sequenced genome data are available to scientific research community. Based on those data, the study of pooling designs has become a very important research direction. Doing research in this direction requires high-quality DNA libraries. A DNA library is a collection of cloned DNA segments, usually from a specific organism. Those cloned DNA segments are called clones. Given a DNA library, the problem is to identify each clone whether it contains a probe from a given set of probes. A probe is a piece of DNA, labeled with radioisotope or fluorescence to identify specific DNA sequences by hybridization. A clone is said to be positive if it contains a given probe, and negative, otherwise. A pool is positive if it contains a positive clone, and negative, otherwise. In a pooling design algorithm, a clone may appear in two or more pools. Hybridization is one of techniques to reproduce clones or to do DNA cloning.

DNA microarray is a solid surface where sequences from thousands of different genes are immobilized, or attached, at fixed locations. DNA microarray is also a tool for performing large numbers of hybridization experiments in parallel. GeneChip [1] is one of DNA microarray technology. It contains a very large number of genes in a small size chip.

DNA microarray technology falls into two applications. We can determine the expression levels of gene-specific probes by observing hybridizations of mRNA to different probes on a microarray. This can be used for drug development, drug response, and therapy development. We can also identify the presence or absence of gene sequences by observing appropriate hybridization reactions. Many domains benefit from this application, such as medicine, environmental science, and industrial quality control.

A biological sample is a biological object such as blood. Targets are for example viruses or bacteria. In order to identify the target sequences in a biological sample, unique probes are preferred since each unique probe only hybridizes to a specific target. In terms of identification, it has strong separability of target sequences. Thus establishing the presence of targets in a sample by using unique probes is obvious and straightforward. Although probes selection is of great importance in many applications, only a few works have been done on this problem. Other than using random probes, some methods choose probes based on their frequencies in the clones [2]. Several methods select probes according to G+C content [3,4] and free energy and melting temperature [5]. This problem has been devised as an optimization problem in [6], in which a greedy heuristic derived from clustering and entropy has been proposed. Furthermore, two heuristics have been proposed for two alternative formulations of the probes selection problem in [7]. One heuristic is based on simulated annealing, which has been proposed for MDPS (Maximum Distinguishing Probe Set) and the other heuristic based on Lagrangian relaxation has been proposed for MCPS (Minimum Cost Probe Set).

In reality, finding unique probes for every target is a difficult task because of the strong similarity of closely related targets, for example, virus subtypes, though temperature and salt concentration are helpful experimental conditions for a probe to uniquely hybridize to its intended target. We need to make use of non-unique probes which hybridize to more than one target in a sample. In terms of non-unique probes, only two works have addressed this problem in literature to the best of our knowledge. A greedy heuristic algorithm has been proposed in [8] to identify the presence or absence of targets in a sample using non-unique probes. Indeed, at first, a 1-separable submatrix is constructed by adding probes one by one, and then a number of pairs of target sets are added randomly to distinguish all those target sets. Therefore, the constructed submatrix is not totally $\bar{d}$-separable. It is the first work that explicitly considers cross-hybridization and experimental errors. This algorithm is simple, practical and time effective. However, the resulting probe set is not guaranteed to be minimal. Moreover, the corresponding decoding algorithm is complicated and time-consuming. An Integer Linear Programming method has been proposed in [9] to reduce the number of probes in the greedy design. This method consists of two ILP formulations. The master ILP guarantees the pairwise separation of all targets, which means all targets are separated by at least *d* probes. The slave ILP guarantees the separation between pairs of small target groups. A cutting plane approach has been proposed to handle the master ILP and the slave ILP. Whenever there is a feasible solution to the master ILP, the slave ILP is applied to check for violated group inequalities which are added to the master ILP to solve it again. This process is iterated until no further violated inequalities are found. The ILP algorithm studies a 1-separable matrix with *k* errors. Therefore, there is no improvement in terms of decoding complexity.

**Figure 1:**
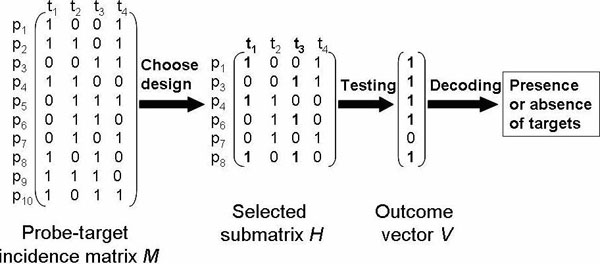
An Example of the 3-steps algorithm

The identification problem of determining the presence of targets in a biological sample using non-unique probes can be solved in three steps suggested by Schliep and Torney [8]: (1) Pre-select suitable probe candidates and compute the probe-target incidence matrix *M *[10,11]. (2) Select a minimal set of probes and compute a suitable design matrix *H* (a submatrix of *M*) to identify up to *d* targets. (3) Decode the presence or absence of targets in a sample from testing outcomes. An example of this algorithm is shown in Figure 1. The rows in *M* represent the set of non-unique probes *p_i_*, and the columns in *M* represent the set of targets *t_j_*. Let *M_ij_* denotes an entry at cell *M*[*i*,*j*]. *M_ij_*=1 if probe *p_i_* hybridize to target *t_j_*; otherwise, *M_ij_*=0. *V* is a test outcome vector. *V_i_*=1 if probe *p_i_* hybridizes to at least one target in the sample; otherwise, *V_i_*=0. In Figure 1, an example of *V* is given when two targets *t*_1_ and *t*_3_ are present in a given sample. A column is called an isolated column iff there is a row containing only one 1-entry at the intersection with that column. In the non-unique probes selection problem, a column (target) *t_j_* is isolated if there is a probe *p_i_* that only hybridizes to one target *t_j_*, that means *p_i_* is a unique probe. Since we only consider non-unique probes, we can assume that the given matrix *M* does not contain any isolated column. Our study focuses on the second step which is called non-unique probes selection. It is concerned about selecting a minimum set of probes while maintaining good decoding ability. Using all probes in *M* always gives the best separation properties of the design. However, some probes have overlapped decoding ability and several probes might hybridize to the same target set so that keeping all of them in the design is not necessary. The number of selected probes is exactly the number of hybridization experiments needed. By selecting a minimum set of probes, we can reduce the number of hybridization experiments, which results in reducing the experimental cost and effort. And also we can have a smaller design which leads to a smaller chip and significant reduction of cost.

While minimizing the number of probes, the decoding ability should not be compromised. The following definitions need to be introduced first. A matrix is a *d*-separable matrix iff the unions of any *d* columns are distinct. The union of *d* columns is the boolean sum of these *d* columns. A matrix is defined as a $\bar{d}$-separable matrix if the unions of at most *d* columns are different. A matrix is a *d*-disjunct matrix iff the union of any*d* columns does not contain any other column. For example, the submatrix *H* in Figure 1 is a 2-disjunct matrix. *d*-disjunct matrices have a decoding complexity of O(*n*), which is much lower than that of *d*-separable matrices, which is O(*n^d^*). We choose to construct a *d*-disjunct matrix *H* instead of a *d*-separable matrix considering the computational complexity of decoding. In this study, we propose efficient non-unique probes selection algorithms using *d*-disjunct matrices to identify the presence of up to *d* targets in a given sample. We also consider the error tolerance case where there are at most *k* experimental errors. The experimental results show that the decoding time is much faster than that of the methods using *d*-separable matrices while running time and solution size are comparable.

## Results and discussion

### The first algorithm

In this section, we study the following problem:

**Problem 1**: MIN-*d*-DS (Minimum *d*-Disjunct Submatrix): Given *m* non-unique probe candidates and a *m* × *n* probe-target incidence matrix *M*, select a minimum set of the probe candidates such that the *h* × *n* submatrix *H* is *d*-disjunct, where *h*≤*n*.

In [12], Du and Hwang have shown that MIN-*d*-DS is NP-hard [13] when *d*=1. Recently, Thai and Znati have shown that MIN-*d*-DS is NP-hard for any fixed *d*≥*1*[14,15]. Due to its hardness, we present a heuristic to approximate the minimum set of non-unique probes selected for the MIN-*d*-DS problem.

The following is another definition for a *d*-disjunct matrix:

**Definition 1**: *H* is a *d*-disjunct matrix iff for any (*d*+1) columns, there exist a row such that an entry at one of those (*d*+1) columns is 1 and entries at the remaining *d* columns are 0.

We assume that a given matrix *M* does not contain any isolated columns since we only consider non-unique probes. The submatrix *H* certainly does not contain any isolated columns either. We have the following definition and lemma:

**Definition 2**: *H* is called (*d*,*k*)-disjunct if for any column *t_j_*,*t_j_* must have at least *k*+1 1-entries not contained in the union of other *d* columns.

**Lemma 1**: Every (*d*+*k*)-disjunct matrix without isolated column is a (*d*,*k*)-disjunct matrix. [12]

We propose an ILPEF (an ILP-based algorithm for the Error Free case) algorithm which consists of two ILP formulations (ILP1 and ILP2) to deal with Problem 1. According to Definition 1, we eliminate those probes that hybridize to at least *n*-*d*+1 targets since they do not contribute to *d*-disjunctness. Then based on Lemma 1, we use ILP1 to construct a (1,*d*-1)-disjunct matrix which facilitate the construction of a *d*-disjunct matrix later. ILP2 is applied to find violations of *d*-disjunctness and those violations are addressed one by one. Finally, the resulting submatrix *H* is *d*-disjunct.

**ILPEF Algorithm**:

1. eliminate all the probes that hybridize to more than *n*-*d*-1 targets

2. select the minimum number of probes *P* to construct a (1, *d*-1)-disjunct matrix using **ILP1**

3. while **(ILP2** finds target sets *R* and *S* which violate *d*-disjunctness)

if there is a probe *p* that hybridizes to the single column in *S* and does not hybridize to any columns in set *R*

P=P U {p}

else

return no solution;

end if

end while

Our formulation of the ILP1 is based on the following definitions. *M* is an *m* × *n* binary matrix with rows representing a set of non-unique probes *p_i_*, ∀i=1,…,m and columns representing a set of targets *t_j_*, ∀j=1,…,n. if probe *p_i_* hybridize to target *t_j_*; otherwise, *M_ij_*=0. *x_i_* is a set of binary variables with *x_i_*=1 if probe *p_i_* is chosen in the submatrix *H* and 0 otherwise. The details of the ILP1 are as follows.

**ILP1 Formulation**:

$$\begin{array}{lll} \min & \sum_{i=1}^{m}x_{i} & \\ & \sum_{i\in M}|M_{ij}-M_{ij}M_{ik}|x_{i}\geq d & \forall j,k\in N,j\neq k\\ & x_{i}\in \left\{0,1\right\} & \forall i\in M \end{array}$$

The first constraint simply guarantees that any column in *M* has at least *d* 1-components not contained in any other column.

After applying the ILP1 on *M*, we have a submatrix which is a (1, *d*-1)-disjunct matrix. In order to construct a *d*-disjunct matrix *H*, we need to find any target sets *R* and *S*(*S* is a singleton set) that violate *d*-disjunctness and add an appropriate probe to cover *R* and *S* until no violation can be found. A probe is said to cover *R* and *S* if this probe hybridizes to the single column in *S* and does not hybridize to any columns in set *R*. The followings are definitions for the formulation of the ILP 2. *w^R^* is the vector that results from the union of columns in target group *R*. *X* is the index set of the currently chosen probes. $X=\{i|x_{i}^{*}=1\},$, x* is a solution vector of the ILP 1. We define $\sigma_{i}^{01}=1.$ iff $w_{i}^{R}=0$ and $w_{i}^{s}=1;$ otherwise, $\sigma_{i}^{01}=0.$*r* and *s* are variable vectors of target groups *R* and *S*. We define *r_j_*=1 if *t_j_* is in group *R* and *s_j_*=1 if *t_j_* is in group *S*. The formulation of the ILP 2 is as follows.

**ILP2 Formulation**:

$$\min\sum_{i\in X}\sigma_{i}^{01}$$

s.t.

(1) $$\begin{array}{cc} \sigma_{i}^{01}\leq 1-r_{j} & \forall i\in X,\forall \end{array}j\in N:M_{ij}=1$$

(2) $$\begin{array}{cc} \sigma_{i}^{01}\leq \sum_{j\in N}M_{ij}s_{j} & \forall i\in X \end{array}$$

(3) $$\begin{array}{cc} \sigma_{i}^{01}\geq \sum_{j\in N}s_{j}-\sum_{j\in N}r_{j} & \forall i\in X:M_{ij}=1 \end{array}$$

(4) $$\sum_{j\in N}s_{j}=1$$

(5) $$\begin{array}{cc} \sum_{j\in N}r_{j}=d & \forall j\in N \end{array}$$

(6) $$r_{j}+s_{j}\leq 1$$

(7) $$\begin{array}{cc} 0\leq \sigma_{i}^{01}\leq 1 & \forall i\in X \end{array}$$

(8) $$r_{j}\in \left\{0,1\right\}\;\forall j\in N$$

(9) $$s_{j}\in \left\{0,1\right\}\;\forall j\in N$$

The first two constraints defines two conditions that $\sigma_{i}^{01}=0.$ Constraint (3) defines the condition that $\sigma_{i}^{01}=1.$ Constraint (4) guarantees that *S* is a singleton set and constraint (5) makes sure that *R* is a set of *d* targets. Constraint (6) keeps *R* and *S* disjoint and the rest constraints are trivial. The ILP2 is repeated until the minimum value found is at least 1, which means no violation of *d*-disjunctness is found.

When there is a violation of *d*-disjunctness, an appropriate probe needs to be added to the design. If there is more than one probe that hybridizes to the single column in *S* and does not hybridize to any columns in set *R*, *p* is selected using a greedy heuristic. We pick the one that covers the most number of (*S*, *R*) pairs. A probe covers a pair (*S*, *R*) when it hybridize to*S* but does not hybridize to any target in *R*. For a probe candidate that hybridizes to *k* targets, the number of covered pairs is $C_{k}^{1}C_{n-k}^{d}.$

After applying our ILPEF algorithm, the resulting submatrix *H* is *d*-disjunct. The decoding algorithm for a *d*-disjunct matrix is simply based on the following lemma:

**Lemma 2**: For testing based on a *d*-disjunct matrix, the number of targets not appearing in any negative results is no more than *d*. [12]

In the decoding algorithm, we just need to remove all columns that appear in the negative results. The remaining columns are the targets that need to be identified in the sample. The time complexity of this decoding algorithm is O(*hn*), where *n* is the number of targets in *M* and *h* is the number of selected probes in*H*.

### The second algorithm

The presence of errors due to the noise of hybridizations complicates the non-unique probes selection problem. Test outcomes may consist of two kinds of experimental errors: false positives and false negatives. We can easily add error tolerance in order to identify at most *d* targets with the presence of at most *k* errors in experiments. To achieve this purpose, we can construct a (*d*, 2*k*)-disjunct matrix [16] and study the following problem:

**Problem 2**: MIN-(*d*, 2*k*)-DS (Minimum (*d*, 2*k*) Disjunct Submatrix): Given *m* non-unique probe candidates and a *m* × *n* probe-target incidence matrix *M*, select a minimum set of the probe candidates such that the *h* × *n* submatrix *H* is (*d*,2*k*) disjunct, where *h*<*n*.

We propose an ILPET (an ILP based algorithm for the Error Tolerance case), which also consists of two ILP formulations (ILP2 and ILP3) to find a solution for problem 2. Same as in the ILPEF algorithm for problem 1, we eliminate those probes that hybridize to at least *n*-*d*+1 targets since they do not contribute to *d*-disjunctness. Then ILP3 is used to select the minimum number of probes *P* to construct a (1, *d*+2*k*–1) disjunct matrix. The ILP2 is then applied to the constructed matrix to find any violations of (*d*, 2*k*) disjunctness. The violations will be addressed and the ILP2 is applied again until all the violations have been addressed. The resulting matrix is a (*d*, 2*k*) matrix. The detailed algorithm is given as follows.

**ILPET Algorithm**:

1. eliminate all the probes that hybridize to more than *n*-*d*-1 targets

2. select the minimum number of probes *P* to construct a (1, *d*+2*k*–1)-disjunct matrix using **ILP3**

3. while (**ILP2** finds target sets *R* and *S* which violate (*d*, 2*k*)-disjunctness, i.e., *S* only contains *v* (*v*<2*k*+1) entries not contained in the union of all the columns in *R*)

if there are 2*k*+1-*v* probes *P*_1_ that all hybridize to the single column in *S* and do not hybridize to any columns in set *R*

$P=P\;U\;P_{1}$

else

return no solution;

end if

end while

The ILP3 is similar to the ILP 1. It is based on the same definitions as the ILP1. The details of ILP 3 are given as follows.

**ILP3 Formulation**:

$$\begin{array}{lll} \min & \sum_{i=1}^{m}x_{i} & \\ & \sum_{i\in M}|M_{ij}-M_{ij}M_{ik}|x_{i}\geq d+2k & \forall j,k\in N,j\neq k\\ & x_{i}\in \{0,1\} & \forall i\in M \end{array}$$

The resulting matrix of the ILPET algorithm is a (*d*, 2*k*) disjunct matrix. Du and Hwang have proven the following lemma:

**Lemma** 3 [16] Every (*d*, 2*k*)-disjunct matrix is a *k*-error-correcting *d*-disjunct matrix. According to lemma 3, our ILPET algorithm can correct at most *k* errors. The decoding is made very simple by the following lemma from [16].

**Lemma** 4 [16] Suppose testing done on a (*d*, 2*k*) – disjunct matrix*H* with at most *k* errors, a target is present iff it appears in at most *k* negative results.

Based on lemma 4, the decoding can be done with time complexity of *O*(*hn*), *h* is the number of probes in the matrix. For each target, we just count the number of negative results containing it. If this number is less than *k*, then this target must be present.

### Experimental result

Our experimental study is carried out on Sun Fire 280R, with Solaris 8 operating system. The ILP software package we utilize is ILOG INC's CPLEX 8.1 [17]. We use Java as the programming language. The program invokes CPLEX functions via a Concert Technology library provided by the CPLEX software.

**Figure 2:**
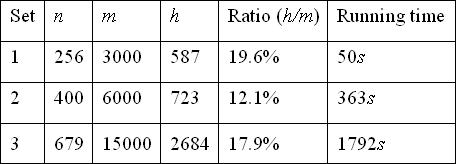
**Results on different datasets. ***n* = # of targets, *m*= # of probe candidates, *h* = # of selected probes

**Figure 3:**
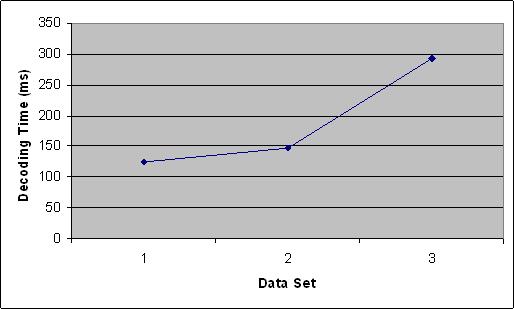
Decoding time for the three datasets

We tested our ILPEF algorithm on three randomly generated datasets, with 3000, 6000, 15000 probe candidates and 256, 400, 679 targets respectively. The result is shown in Figure 2. From the figure, we can see that the absolute running time of our ILPEF approach for the randomly generated datasets is in the range of 50 to 1800 seconds, which is comparable to the running time of Klau's ILP approach [9]. The number of candidates chosen by our ILPEF approach is between 12% and 20%, which is also comparable to Klau's approach. In our experiments, 9.3% of the constructed (1, *d*–1) matrices from the ILP1 are already *d*-disjunct matrices, which saves the effort to run the ILP2 iteratively. Moreover, our ILPEF approach has much faster decoding time. As shown in Figure 3, all decoding operations take less than 1 second. All the decoding results are correct, as expected.

In summary, we chose to generate a *d*-disjunct matrix using our ILPEF approach for the simplicity of decoding. Our experimental study shows that our ILPEF approach can greatly reduce the decoding time while maintain comparable running time and solution size compared with previous approaches, which achieves our design goal.

## Conclusions

A minimization problem arises from the study of non-unique probes selection. However, it is misleading to minimizing the number of probes regardless of the decoding ability of the corresponding design. Efficient non-unique probes selection algorithms for DNA microarray design should reduce the number of probes, which results in the reduction of experimental cost and time, while maintain good decoding ability. In addition, the designed algorithms should be error tolerant since errors are very common in biology experiment.

We have presented solutions for two cases, error free and error tolerance. For each case, we have shown efficient algorithms to select a minimum number of probes as well as to decode the results. These algorithms are able to identify at most *d* targets in a given sample. Our non-unique probes selection can also identify the presence of at most *d* targets in a case of at most *k* experimental errors. In addition, we have also analyzed the running time of how to decode the test results obtained from the microarrays experiments. The time complexity of our decoding algorithms is O(*hn*) where *h* is the number of selected probes. Experimental study has been carried out for the ILPEF algorithm and the results have shown that our ILPEF algorithm can greatly reduce the decoding time of existing approaches while maintain comparable running time and solution size.

Note that *d*-disjunct matrices have a stronger property than that of* d*∀i=1,…,m-separable matrices. Therefore, a* d*-disjunct matrix should have more rows (probes) than that of ∀i=1,…,m-separable matrix. That is a trade-off for having a faster decoding algorithm. We choose to construct a *d*-disjunct matrix which results in a very simple decoding algorithm.

## Methods

### Overall design goals

Since the number of selected non-unique probes is equal to the number of hybridization experiments, we need to find a minimum number of selected probes in order to reduce the experimental cost and time. Furthermore, the selected probes must result in an efficient decoding algorithm, which is used to infer the test outcomes. Finally, the designed algorithms must be robust in order to handle the experimental errors due to noises.

### Integer Linear Programming (ILP)

Integer linear programming involves the optimization of a linear objective function, subject to a number of linear equality or inequality constraints. All the variables in ILP can only take integer values. ILP has been widely used for optimization problems.

Our design is to build a matrix that is close to a *d*-disjunct matrix using one ILP. Then we can formulate another ILP to find out violations of *d-*disjunctness. The violations are addressed and the other ILP is formulated to find out further violations. This goes on until all the violations have been addressed. Then we can obtain a *d*-disjunct matrix. Similar ideas apply to construct a (*d*, 2*k*)-disjunct matrix.

### Experimental data generation

The datasets used for the experimental study are randomly generated. We generate three different datasets, with 3000, 6000, 15000 probe candidates and 256, 400, 679 targets respectively. The values are picked to facilitate comparison with previous approaches and future comparison with real biological datasets. Each candidate hybridizes to a random number of targets. This random number is set to be between *d* and *n* –*d*. *d* is set to be 5, which is the same as in [9]. Measures are taken in the data generation program to make sure that no two probe candidates hybridize to the same set of targets. The result vectors are also generated randomly. Up to *d* targets are randomly picked and the union of those columns is the result vector that can be used to verify the correctness of the decoding process.

## Competing interests

The authors declare that they have no competing interests.

## Authors' contributions

All authors contributed to the design and the writing of the manuscript. All authors read and approved the final manuscript.
